# MiR-29a Inhibits Cell Proliferation and Induces Cell Cycle Arrest through the Downregulation of p42.3 in Human Gastric Cancer

**DOI:** 10.1371/journal.pone.0025872

**Published:** 2011-10-05

**Authors:** Yun Cui, Wen-Yu Su, Jing Xing, Ying-Chao Wang, Ping Wang, Xiao-Yu Chen, Zhi-Yong Shen, Hui Cao, You-Yong Lu, Jing-Yuan Fang

**Affiliations:** 1 Division of Gastroenterology and Hepatology, Shanghai Jiao-Tong University School of Medicine Renji Hospital, Shanghai Institute of Digestive Disease, Shanghai, China; 2 GI Division, No.9 People’s Hospital, Shanghai Jiaotong University School of Medicine, Shanghai, China; 3 GI Surgical Division, Shanghai Jiaotong University School of Medicine Renji Hospital, Shanghai, China; 4 Laboratory of Molecular Oncology, Beijing Institute for Cancer Research, School of Oncology, Peking University, Hai-Dian District, Beijing, China; University of Barcelona, Spain

## Abstract

As a newly identified and characterized gene, p42.3 is associated with cell proliferation and tumorigenicity. The expression of p42.3 is upregulated in human gastric cancer (GC), but its underlying mechanisms of action are not well understood. MicroRNAs (miRNAs) are known to play vital regulatory roles in many cellular processes. Here we utilized bioinformatics and experimental approaches to investigate the regulatory relationship between miRNAs and the p42.3 gene. We showed that miR-29a could repress p42.3 expression at both the mRNA and protein levels via directly binding to its 3’UTR. Furthermore, an inverse relationship was observed between miR-29a and p42.3 expression in gastric cancer cell lines and GC tissue samples, especially in cases where p42.3 was downregulated. Taken together, we have elucidated previously unrecognized roles of miR-29a and indicated that miR-29a may function, at least partially, by targeting the p42.3 gene in human GC.

## Introduction

p42.3 is a novel gene that has been recently isolated and identified by the mRNA differential display (mRNADD) technique. The full-length cDNA of p42.3 is approximately 4.0 kb, and the gene encodes a 389 amino acid (aa) protein that is estimated to have a molecular mass of 42.3 kDa. Further research has revealed that its expression is cell cycle-dependent in gastric cancer (GC) cell lines. Its protein expression peaks during the M phase of the cell cycle, before gradually decreasing after cell division; this indicates that p42.3 may be involved in cell cycle regulation. Furthermore, silencing of p42.3 by small interfering RNA (siRNA) results in the upregulation of CHK2 and the downregulation of cyclin B1, which are two key proteins involved in cell cycle regulation [1], [2]. While RT-PCR and immunohistochemical analyses have shown that p42.3 is upregulated in GC compared with normal tissue samples, functional research has suggested that the depletion of p42.3 may not only result in the inhibition of GC cell proliferation and colony formation *in vitro*, but may also significantly reduce tumorigenicity in nude mice [3]. Although previous studies have suggested a critical role for the p42.3 gene in the pathology of GC, the specific underlying mechanisms of its action remain to be clarified.

MicroRNAs (miRNAs) consist of a class of small (∼22 nucleotides), endogenous, non-coding RNAs that are known to play important regulatory roles in gene expression [4]. The primary miRNA transcript is called pri-miRNA [5], which is transcribed by RNA polymerase II or III [6], [7]. The pri-miRNA is then cleaved by the Drosha-DGCR8 microprocessor complex to produce the precursor hairpin molecule (pre-miRNA) which is then exported from the nucleus to the cytoplasm by exportin−5/Ran-GTP. With the assistance of a complex that contains the RNase Dicer and the double-stranded RNA-binding protein, TRBP, the ∼70-nucleotide pre-miRNA is processed into mature miRNA [8]. The functional strand of the mature miRNA is loaded into the RNA-induced silencing complex (RISC), which contains the proteins, argonaute (Ago) and Tnrc6, while the other strand is usually degraded [9]. The mature miRNA guides the RISC to the imperfect complementary sequences in target mRNAs to repress the cognate mRNA translation, promote transcript decay, or both [10]. It is estimated that most coding genes are probably regulated by miRNAs and, whilst a miRNA may regulate more than one target genes, certain genes can be regulated by multiple miRNAs [11].

Growing evidence suggests that miRNAs are involved in a wide range of physiological and pathological processes, including development, differentiation, proliferation and apoptosis [12.13,14,15]. Although abnormalities of miRNA expression have been determined in many human tumors, including colorectal, gastric and breast cancers [16], [17], the number of such tumors is still expanding. However, the detailed functions of miRNA in tumors remain to be elucidated.

Recent studies have suggested that miR-29 has complex functions in various diseases. MiR-29a may behave as a tumor suppressor in both lung and pancreatic cancer cell lines, and thus the exogenous overexpression of miR-29a results in a significant reduction in the invasive potential and proliferation of these cell lines [18]. The tumor suppressor role of miR-29a is also supported by its observed downregulation in a broad spectrum of solid tumors, including neuroblastoma, sarcomas and brain tumors [19]. In contrast, miR-29a is upregulated in indolent human B-cell chronic lymphocytic leukemia (B-CLL) [20] and acute myeloid leukemia (AML) [21], which suggests a possible tumor promoter role. In addition, the aberrant expression of miR-29a can be found in many non-malignant diseases, including liver fibrosis [22], diabetes [23] and Alzheimer’s disease [24]. Although many genes have already been confirmed to be the direct targets of miR-29a, such as PPM1D [25], PI3K [26] and neuron navigator 3 [24], they represent a very small fraction of the total genes that miR-29a targets.

In the present report, we demonstrate that p42.3 expression was controlled at the levels of both mRNA and protein by miR-29a via direct targeting of the 3’UTR of p42.3. MiR-29a could suppress cell proliferation and induce cell cycle arrest, at least in part, via the downregulation of p42.3 expression. Moreover, we found that the expression of p42.3 protein was inversely correlated with miR-29a expression in human GC tissues.

## Results

### p42.3–3’UTR is putatively targeted by miR-29a

**Figure 1:**
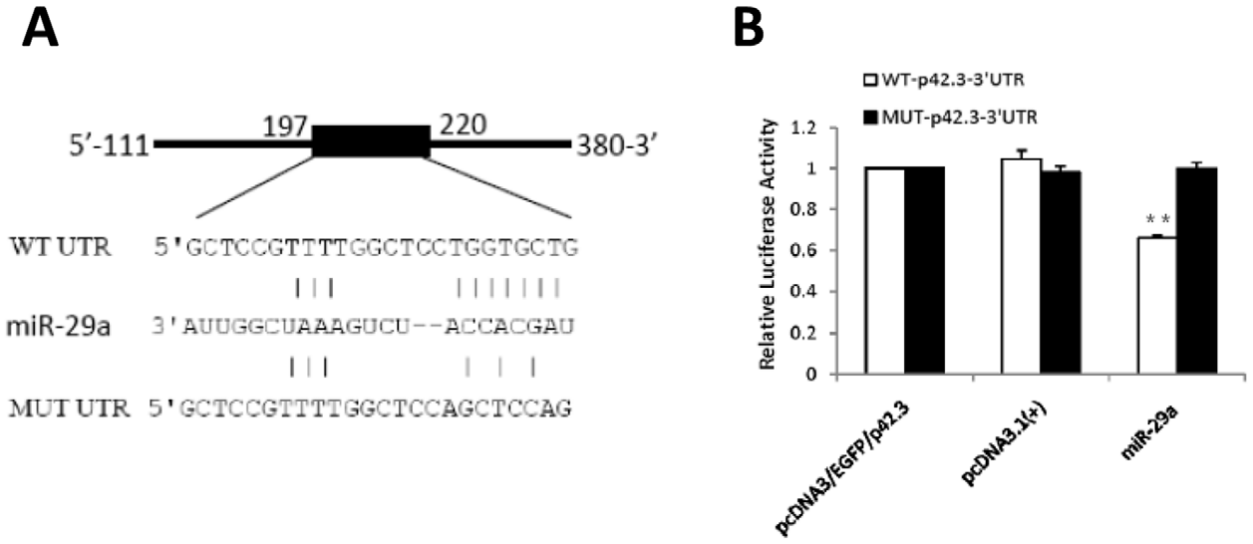
MiR-29a targets a putative binding site in p42.3–3’UTR. **A.** The sequence of miR-29a with the putative binding site in the human p42.3 gene. The putative binding site with the mutant is shown in the lower panel. **B.** Regulation of reporter gene expression by miR-29a in MKN-45 cells co-transfected with the pri-miR-29a and reporter gene containing the putative binding site. **: *P*<0.01.

Putative miRNAs that were predicted to target the p42.3 gene by more than one database were analyzed. The top two miRNAs which were predicted for three times were selected for further confirmation and the other three miRNAs, including miR-29a, whose putative binding sites were close to those of the top two were also selected as candidates for validation. MiR-29a was predicted by the TargetScan and miRGEN databases, which indicated that its putative binding site was at positions 213–219 of the p42.3–3’UTR (Figure 1A). The other candidates are not listed here.

### Directly targeting the p42.3–3’UTR by miR-29a

The reporter gene assay was employed to validate whether p42.3 was a direct target of miR-29a. Wild-type and mutant p42.3–3’UTR containing the putative binding site of miR-29a were cloned into individual plasmids and fused with the reporter gene. The fluorescent intensity of the reporter gene was significantly decreased in the group that was co-transfected with WTpcDNA3/EGFP/p42.3 and pcDNA3.1/pri-miR-29a compared to the control. In addition, there was no significant decline of the fluorescent intensity in the group that was co-transfected with MUTpcDNA3/EGFP/p42.3 (Figure 1B), further confirming that miR-29a induced the downregulation of p42.3 gene expression via the specific binding of the putative site of the p42.3–3’UTR.

### Expression of miR-29a and p42.3 are inversely related in GC cell lines

**Figure 2:**
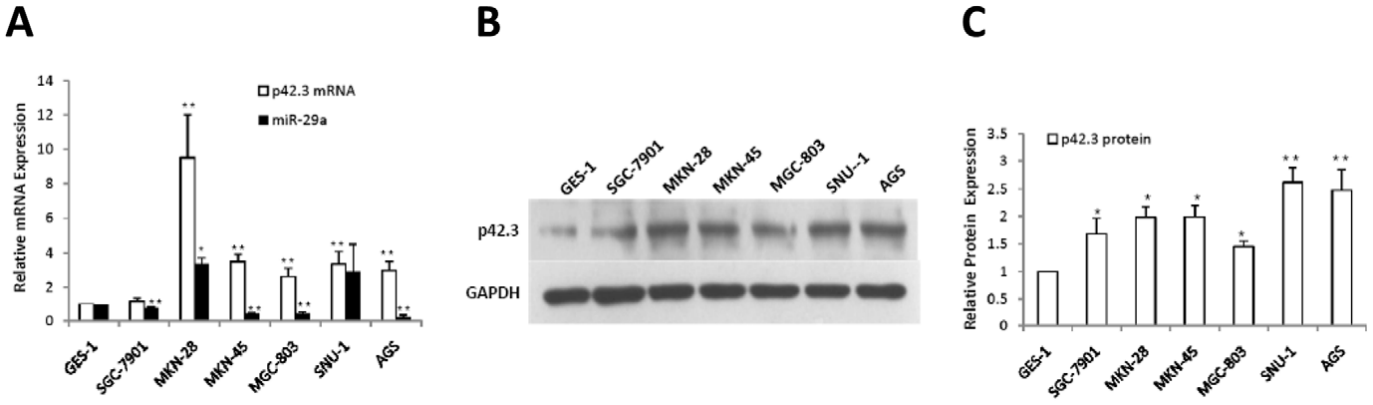
Expression of endogenous p42.3 and miR-29a in GC cells. **A.** Expression of p42.3 mRNA and mature miR-29a in six GC cell lines (SGC-7901, MKN-28, MKN-45, MGC-803, SNU-1, and AGS) normalized to the normal gastric epithelium cell line (GES-1) using quantitative real-time RT-PCR. Endogenous references were GAPDH and U6 small nuclear RNA, respectively. *: *P*<0.05, **: *P*<0.01. **B and C.** Expression of p42.3 protein in GC and normal gastric epithelial cell lines analyzed by western blotting (B) and shown as mean ± SD (normalized; C). *: *P*<0.05, **: *P*<0.01.

To ascertain the link between p42.3 and miR-29a, we studied the endogenous expression of miR-29a and p42.3 in six human GC cell lines (SGC-7901, MKN-28, MKN-45, MGC-803, SNU-1 and AGS) and one normal gastric epithelium cell line (GES-1; Figure 2A–C). We found that the level of p42.3 mRNA was significantly higher than the normal control in all GC cell lines with the exception of SGC-7901, where the difference was not statistically significant. The expression level of p42.3 was variable at the protein level, but was significantly higher in all of the GC cell lines when compared with GES-1.We also showed that miR-29a expression was low in four of the GC cell lines (SGC-7901, MKN-45, MGC-803 and AGS), which revealed an inverse relationship with p42.3 expression. Although the expression of miR-29a was high in SNU-1, this was not statistically significant. It is worth mentioning that the high expression of miR-29a in MKN-28 was an exception.

### MiR-29a regulates p42.3 expression

**Figure 3:**
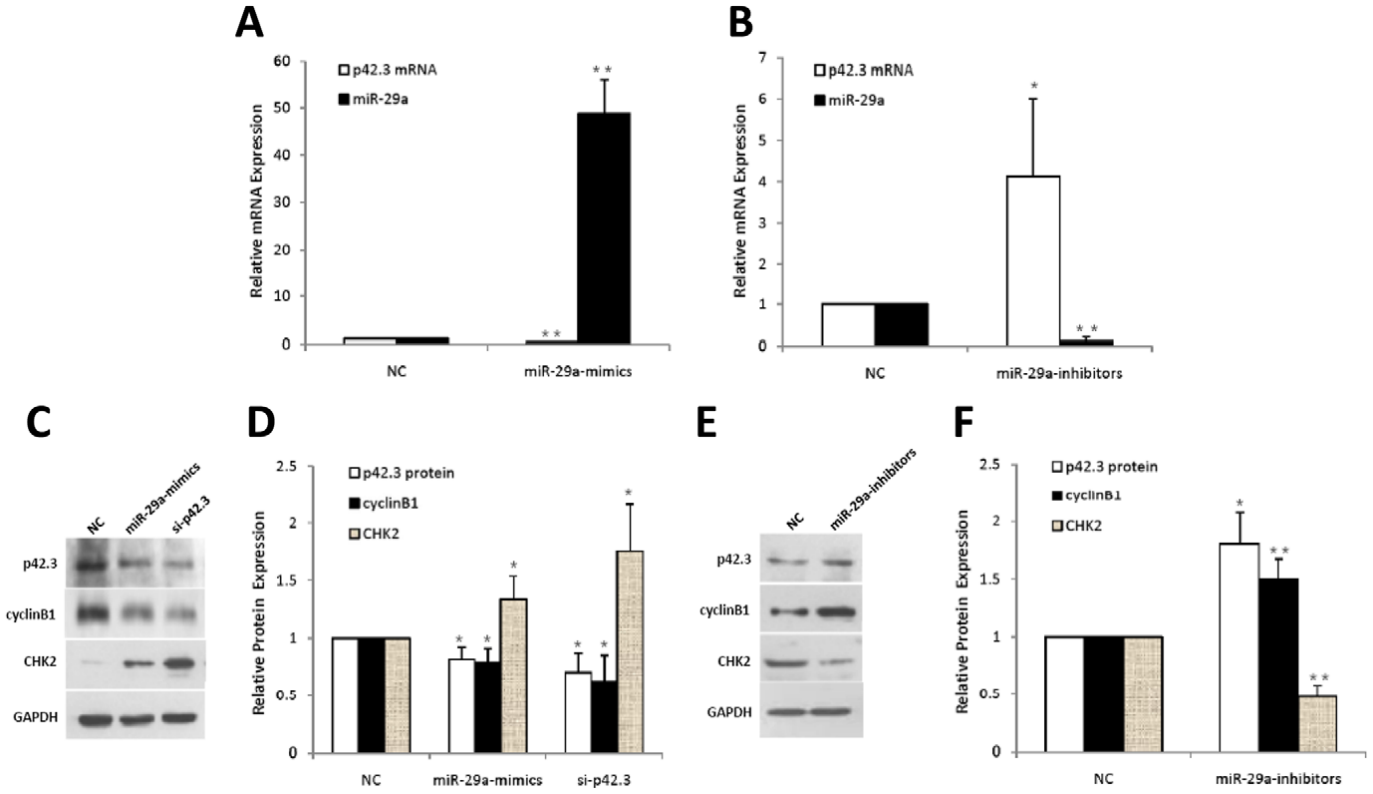
Regulation of endogenous p42.3 expression by miR-29a. **A.** Effect of miR-29a mimics on the endogenous expression of p42.3 mRNA. Mature miR-29a was exogenously upregulated while the endogenous expression of p42.3 mRNA was significantly downregulated in MKN-45 cells. **: *P*<0.01. **B.** Effect of miR-29a inhibitors on the endogenous expression of p42.3 mRNA. Mature miR-29a was exogenously downregulated, while the endogenous expression of p42.3 mRNA was significantly upregulated in MKN-45 cells. *: *P*<0.05, **: *P*<0.01. **C and D.** Effect of miR-29a mimics on the endogenous expression of p42.3, cyclin B1 and CHK2 proteins were similar to those of si-p42.3. The expression levels of p42.3 and cyclin B1 protein decreased significantly after MKN-45 cells were treated with si-p42.3 or miR-29a mimics while the level of CHK2 protein increased dramatically. Data are shown as mean ± SD (normalized; D). *: *P*<0.05. **E and F.** Effect of miR-29a inhibitors on the endogenous expression of p42.3, cyclin B1 and CHK2 protein. MiR-29a inhibitors reversed the expression changes seen in C and D. *: *P*<0.05, **: *P*<0.01.

To evaluate whether p42.3 was repressed by miR-29a, we treated MKN-45 cells with the mimics and inhibitors of miR-29a for 48 h in order to exogenously up- and downregulate the expression of miR-29a specifically; the expression of p42.3 was then determined. After the transfection of MKN-45 cells with the mimics, the miR-29a expression increased by approximately 10-fold, while the treatment with the inhibitors decreased the miR-29a level by more than 50% (Figure 3A–B). This suggested that both the mimics and inhibitors of miR-29a worked efficiently in our experiments. Overexpression of miR-29a could significantly repress the expression of p42.3 at both the mRNA and protein levels, which was a similar effect to the silencing of p42.3 by p42.3 siRNA (si-p42.3). We also detected that CHK2 was upregulated and cyclinB1 was downregulated after the silencing of p42.3 by p42.3 siRNA. Interestingly, similar effects were exerted on CHK2 and cyclinB1 by the overexpression of miR-29a in MKN-45 cells (Figure 3C–D). In addition, transfection of miR-29a inhibitors dramatically decreased CHK2 expression and increased p42.3 and cyclinB1 expression (Figure 3E–F). This suggested that miR-29a could regulate the expression of the p42.3 gene in MKN-45 cells.

### MiR-29a inhibits cell proliferation in vitro

**Figure 4:**
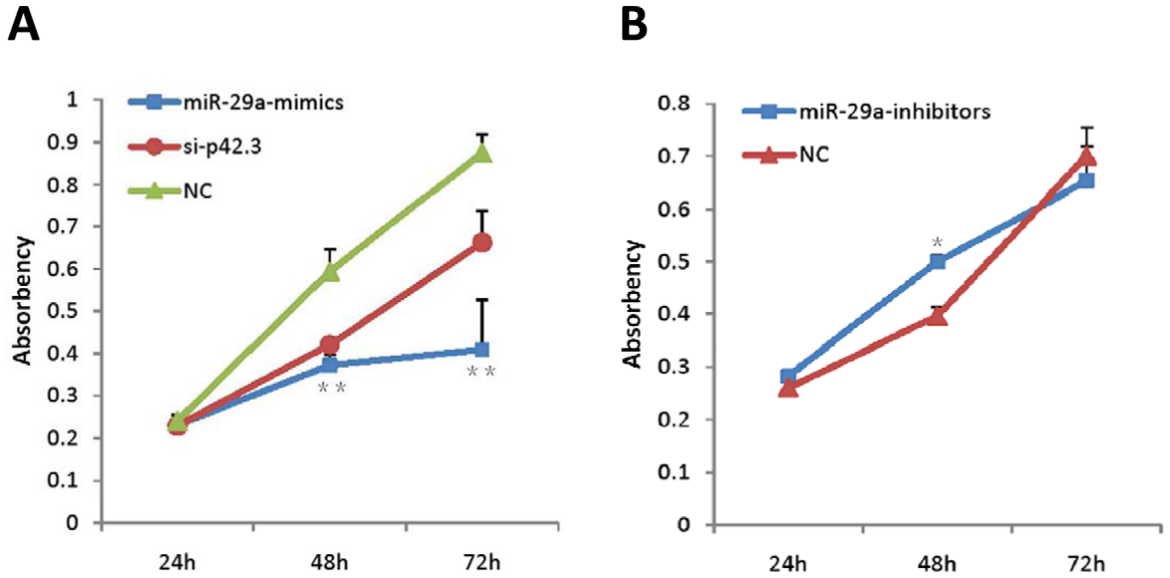
Effect of miR-29a on cell proliferation in the MKN-45 cell line. **A.** MiR-29a mimics inhibited cell proliferation and the inhibiting rate at 48 h was approximately 20% and approximately 40% at 72 h, while p42.3 siRNA inhibited cell proliferation by approximately 15% at 48 h and 72 h. **: *P*<0.01. **B.** MiR-29a inhibitors promoted cell growth by approximately 10% at 48 h, but the effect weakened at 72 h. *: *P*<0.05.

According to the data of the cell proliferation assay, we drew the absorbency curves at the wavelength of 450 nm after transfection for different durations. We found that cell proliferation was significantly inhibited after the transfection of MKN-45 cells with p42.3 siRNA for 48 h and 72 h, and a similar pattern was noted after cells were transfected with mimics of miR-29a. However, the degree of repression achieved by miR-29a mimics was greater than p42.3 siRNA-induced downregulation (Figure 4A). On the contrary, cell growth was promoted by approximately 10%, compared to the negative control when transfected with inhibitors of miR-29a for 48 h, but there was a decline at 72 h (Figure 4B). This indicated that miR-29a could inhibit cell proliferation via repressing the expression of p42.3.

### MiR-29a blocks cell cycle progression

**Figure 5:**
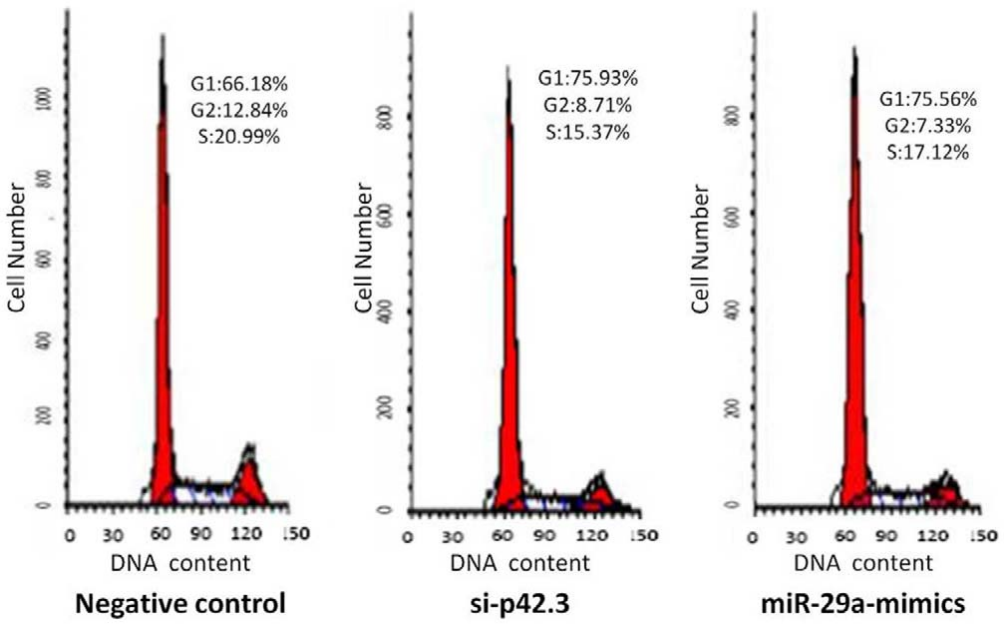
Effect of miR-29a on cell cycle in the MKN-45 cell line. Flow cytometric analysis confirmed that both p42.3 siRNA and miR-29a mimics induced G1 phase arrest in the MKN-45 cell line.

Silencing of p42.3 by p42.3 siRNA can result in cell cycle arrest; therefore, we investigated whether miR-29a could affect the cell cycle progression via targeting the p42.3 gene. After MKN-45 cells were transfected with p42.3 siRNA for 48 h, we found that the cell cycle was blocked at the G1 phase (75.93%, *P*<0.05), compared with the negative control (66.18%). We found that mimics of miR-29a could also induce G1 phase arrest in MKN-45 cells when treated for 48 h (75.56%, *P*<0.05; Figure 5).

### Expression of miR-29a and p42.3 protein in GC and their correlation with clinicopathological characteristics

**Figure 6:**
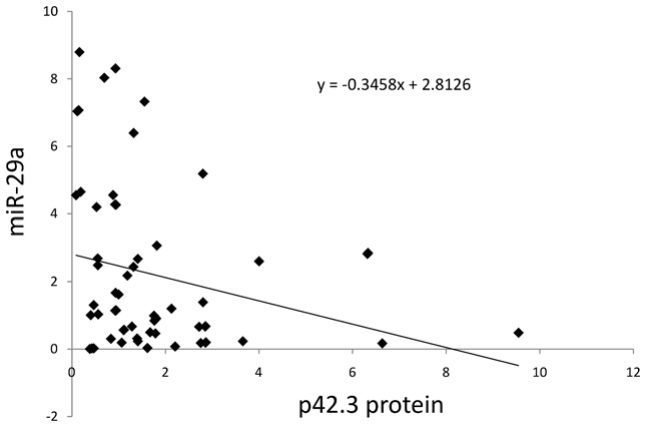
The scatterplot of the expression of p42.3 protein and miR-29a in 60 gastric cancer tissues. There was an inverse relationship between the expression of p42.3 protein and miR-29a and the equation of their relationship was y = −0.3458x+2.8126 (y: the expression of miR-29a, x: the expression of p42.3 protein).

Using a quantitative real-time PCR technique, miR-29a was detected in 60 pairs of GC tissues and their matched non-cancer adjacent tissues, while p42.3 protein level was also evaluated in these tissues by Western blotting. Out of 60 GC tissues samples, p42.3 expression was high in 35 cases (35/60, 58.33%) relative to their matched non-cancer adjacent tissues. In the 25 cases in which p42.3 expression was downregulated, miR-29a expression was high in 21 cases. MiR-29a expression was low in 27 cases (27/60, 45%). The above data suggests an inverse relationship between miR-29a and p42.3 protein expression in tissue samples (*P* = 0.000, Table 1 and Figure 6), but the correlation coefficient was not good (r = −0.316).

Furthermore, miR-29a and p42.3 protein expression were evaluated with regards to the clinicopathological characteristics of the 60 patients from whom tissue samples were taken. Our findings suggested that there were no obvious correlations between p42.3 protein and miR-29a expression, respectively, with clinicopathological features (Table 2).

**Table 1:** Correlation between the expression of p42.3 protein and miR-29a in 60 gastric cancer tissues.

	miR-29a				Spearman's correlation	
	low	high	n	P	P	r
**p42.3**						
low	4	21	60	0.000	0.014	−0.316
high	23	12				

**Table 2:** Associations between the expression of p42.3 protein and miR-29a with clinicopathological features of 60 patients with gastric cancer.

	n	p42.3^a^	miR-29a^a^
**Sex**			
Male	37	1.06(0.55−1.67)	0.99(0.23−2.69)
Female	23	1.79(0.97−2.78)	1.39(0.78−2.96)
**P**		0.712	0.258
**Age**			
≤65	36	1.25(0.87−1.77)	1.35(0.55−4.26)
>65	24	1.53(0.56−2.86)	0.89(0.29−2.52)
**P**		0.811	0.19
**Histologic grade**			
Good	15	1.75(0.68−2.34)	0.46(0.20−2.03)
Poor	45	1.19(0.68−2.34)	1.30(0.67−2.85)
**P**		0.906	0.374
**pT stage**			
T1+2	8	0.62(0.49−0.97)	1.49(0.31−5.16)
T3+4	52	1.36(0.93−2.73)	1.09(0.54−2.85)
**P**		0.199	0.984
**pN stage**			
N0	14	1.06(0.48−1.76)	0.58(0.31−2.85)
N1	21	1.06(0.93−2.80)	1.62(0.68−4.26)
N2	12	1.15(0.62−1.57)	1.69(0.56−7.07)
N3	13	1.79(0.95−2.75)	0.91(0.66−1.15)
**P**		0.351	0.359
**pTNM stage**			
I	7	0.56(0.46−1.11)	0.50(0.31−3.34)
II	6	1.55(0.67−5.20)	1.76(0.18−2.85)
III	32	1.15(0.92−2.15)	1.50(0.54−4.34)
IV	15	1.79(0.95−2.74)	1.01(0.78−1.15)
**P**		0.138	0.711

a Median of relative expression, with 25 th–75 th percentile in parenthesis.

## Discussion

The p42.3 gene is highly conserved in mammals and, as an oncogene, it may play an important role in the progressive transformation of normal gastric epithelium cells to cancer cells. Its differential expression during the cell cycle stages reveals that p42.3 may be involved in cell cycle regulation. This was further confirmed by our results, which showed that p42.3 silencing could alter the expression of two key proteins, CHK2 and cyclin B1, that are involved in cell cycle regulation. Using loss-of-function experiments, we demonstrated that p42.3 may stimulate cellular proliferation and as reported, our results showed that p42.3 was overexpressed in GC tissues when compared with the adjacent non-cancer mucosa [3]. However, the molecular mechanisms resulting in this aberrant expression of p42.3 gene in GC is poorly understood, as evident from the lack of available literature. MiRNAs can regulate gene expression by targeting the binding sites in the target mRNAs [27] and, in human cancers, many miRNAs have already been implicated. However, the function of only a few has been understood to date, especially in GC [28].

In this report, we selected miR-29a for further investigation by a reporter gene system and ultimately identified that p42.3 was a direct target gene of miR-29a. Our data suggested that the four databases (TargetScan, microRNA.org, MicroCosm Targets Version 5 and miRGen) used were efficient, but not perfect, tools for the prediction of miRNA targets and provided experimental evidence for improvements to the underlying algorithm.

We showed that p42.3 expression was inversely related with miR-29a expression in four GC cell lines. In three of these, MKN-45, MGC-803 and AGS, this was evident at both the mRNA and protein level, but this was not the case in the SGC-7901 cell line. In addition, miR-29a expression was not low in the MKN-28 and SNU-1 cell lines. Taken together, these observations allowed us to hypothesize that the regulatory role of miR-29a is complicated in GC cell lines and that miR-29a may play different roles in different cellular backgrounds. However, the detailed mechanisms of miR-29a functions in GC cell lines require further investigation.

In our study, miR-29a overexpression could induce similar effects to those achieved by the silencing of p42.3 by p42.3 siRNA. Both of p42.3 mRNA and protein were downregulated after cells were transfected with p42.3 siRNA or miR-29a mimics and the cell proliferation and cell cycle were suppressed when cells underwent the same interference. It suggested that miR-29a may be involved in the pathogenesis of GC via the repression of p42.3 gene expression. However, we found from the growth curves that the degree of repression by miR-29a mimics was greater than by p42.3 siRNA. In our opinion, the main reason for this may be that specific miRNAs could regulate hundreds of genes to control cellular function. In the present study, miR-29a may inhibit cell proliferation, at least in part, by targeting p42.3.

Our data showed that although the rate of high expression of miR-29a was 55% (33/60) in GC, p42.3 expression was low in 21 of these cases. This suggested that miR-29a may have a vital role in GC tissues with low expression levels of p42.3.

In the present study, we validated that p42.3 is a direct target of miR-29a. We have also demonstrated that miR-29a can inhibit cell proliferation and block the cell cycle, at least in part, via the repression of p42.3 expression in GC. We conclude that miR-29a may play a critical role in regulating the expression of p42.3 in GC. Interestingly we found that p42.3 gene could also regulate the expression of miR-29a (the data are not shown here), but the underlying regulatory mechanism will be explored in the future.

## Materials and Methods

### Bioinformatics

Four efficient computational approaches that utilize different evaluating systems [29]–[32] were used for the prediction of the regulatory miRNAs that target the p42.3 gene, including TargetScan (http://www.targetscan.org/), microRNA.org (http://www.microrna.org/microrna/getGeneForm.do/), MicroCosm Targets Version 5 (http://www.ebi.ac.uk/enright-srv/microcosm/htdocs/targets/v5/) and miRGen (http://www.diana.pcbi.upenn.edu/cgi-bin/miRGen/v3/Targets.cgi#Results). Targets were selected for further confirmation from the group of miRNAs that were common to the results generated from more than one search.

### Tissue samples

Sixty pairs of histopathologically confirmed GC and adjacent non-cancer tissue samples were obtained from patients who underwent surgical resection at the Renji Hospital affiliated to the Shanghai Jiaotong University School of Medicine, China between July 2007 and January 2009. The matched non-cancer adjacent tissues were obtained at least 5 cm away from the tumor site. The study was approved by the Research Ethics Committee of Shanghai Jiaotong University and informed consent was obtained from all patients, while written consent were obtained from each patient.

### Cell lines and culture conditions

Human GC cell lines, SGC-7901, MKN-28, MKN-45, MGC-803, SNU-1 and AGS, and the GES-1 normal gastric epithelium cell line, which were purchased from ATCC (USA), were maintained in RPMI 1640 medium (Gibco, Gaithersburg, MD, USA) supplemented with 10% fetal bovine serum (Invitrogen, Carlsbad, CA, USA). Cells were cultured at 37°C in a 5% CO_2_ incubator. A solution of trypsin (0.25%) was used to detach the cells from the culture flask.

### Vector construction

To construct the expression vectors pri-miR-29a was first amplified with primers designed by Primer Premier 5.0 software (Table 3) and then cloned into pcDNA3.1 (Invitrogen). To produce the plasmids that contained the putative binding site of miR-29a, both wild-type and mutant sequences from position 111 to 380 of the p42.3–3’UTR were chemically synthesized (Figure 1A) and then were cloned to the downstream of the EGFP gene at *Bam*HI and *Eco*RI sites in the pcDNA3/EGFP vector (Saierbio, Tianjin, China).

**Table 3:** Primer sequences of the related genes.

name	primer sequence
Pri-miR-29a-sense	5'-CGCGGATCCTGGATTTAGTAAGATTTGGGC-3'
Pri-miR-29a-anti	5'-CCGGAATTCACATGCAATTCAGGTCAGTG-3'
p42.3-mRNA-sense	5'-CCTGGCATCTTTACTGGACTGGA-3'
p42.3-mRNA-anti	5'-GTGCCAGCCTGTCTCACATTTC-3'
GAPDH-mRNA-sense	5'-CACCATCTTCCAGGAGCGAG-3'
GAPDH-mRNA-anti	5'-GGGGCCATCCACAGTCTTC-3'

### Reporter gene assay

MKN-45 cells were seeded in triplicate wells of a 24-well plate on the day before transfection. PcDNA3.1 (+)/ primary-miR-29a were co-transfected with wild-type and mut-pcDNA3/EGFP/p42.3–3’UTR respectively. Then, 0.5 µg of pcDNA3.1 (+)/ primary-miR-29a and 0.5 µg of pcDNA3/EGFP/p42.3–3’UTR were added into each of the wells, while 0.1 µg of vector pDsRed-C1 (Clontech), which expressed the RFP, were added to every well as an endogenous reference. Cells that were only transfected with pcDNA3/EGFP/p42.3–3’UTR or pcDNA3/EGFP/p42.3–3’UTR+ pcDNA3.1 (+) were used as the controls. Cells were collected 48 h after transfection and analyzed based on the intensity of EGFP and RFP fluorescence detected using Fluorescence Spectrophotometer F-4500 (Hitachi, Japan).

### Cell transfection

Transfection of MKN-45 cells was performed using the Lipofectamine 2000 Reagent (Invitrogen) following the protocol of the manufacturer. MKN-45 cells were seeded into 6-well plates or 96-well plates at 30% confluence the day prior to the transfection. Mimics (100 nM, GenePharma) and inhibitors (200 nM, RIBOBIO) of miR-29a were used to exogenously up- and downregulate the expression of miR-29a. To silence p42.3 gene expression, the MKN-45 cells were transfected with siRNA against p42.3 (si-p42.3, 100 nM, GenePharma). The control RNA (named as NC) was non-homologous to any human genome sequence. The sequences of the oligo nucleotides are shown in Table 4.

**Table 4:** Sequences of the related oligo nucleotides.

name	sequence
miR-29a-mimics-sense	5'-UAGCACCAUCUGAAAUCGGUUA-3'
miR-29a-mimics-anti	5'-ACCGAUUUCAGAUGGUGCUAUU-3'
p42.3 siRNA-sense	5'-CACUGUCCCACAAGGCACCdTdT-3'
p42.3 siRNA-anti	5'-GGUGCCUUGUGGGACAGUGdTdA-3'
NC-sense	5'-UUCUCCGAACGUGUCACGUTT-3'
NC-anti	5'-ACGUGACACGUUCGGAGAATT-3'
miR-29a-inhibitors	5'-mU(s)mA(s)mAmCmCmGmAmUmUmUmCmAmGmAmUmGmGmUmG(s)mC(s)mU(s)mA(s)-Chol-3'
NC-inhibitors	5'-mC(s)mA(s)mGmUmAmCmUmUmUmUmGmUmGmUmAmGmUmAmC(s)mA(s)mA(s)mA(s)-Chol-3'

### Real-time RT-PCR

Total RNA was isolated using Trizol reagent (Invitrogen) and subsequently treated with RNase-free DNase I (Fermentas, San Diego, CA, USA). Total RNA (500 ng) was reverse transcribed using the PrimeScript RT reagent Kit (TaKaRa, Dalian, Japan). The primer sequences used to amplify p42.3 and GAPDH are shown in Table 3. To analyze the expression of the mature miR-29a, 2 µg of the total RNA was subjected to reverse transcription using the All-in-One MiRNA Q-PCR Detection Kit (GeneCopoeia, Guangzhou, China). Quantitative real-time PCR for mature miR-29a and U6 was performed according to the manufacturer’s instructions using the ABI 7300 real-time PCR system and specific primers designed by GeneCopoeia, China. The relative expression of p42.3 and miR-29a was normalized to an endogenous reference (GAPDH and U6 small nuclear RNA respectively) and relative to the control. The results were presented as fold change, calculated using the 2(−ΔΔCT) method [33]; a relative expression ratio of <1.0 was considered as low, while a ratio of >1.0 was considered as high expression [14].

### Western blotting

Total protein from cultured cells and tissues were extracted by RIPA Lysis Buffer containing PMSF, according to the manufacturer’s instruction (Beyotime, Shanghai, China). Protein concentration was measured using the Bradford method [34]. Overall, 40 µg of protein were electrophoresed through 10% SDS polyacrylamide gels and were then transferred to a PVDF membrane (Millipore). The membrane was incubated with p42.3 antibody (1∶500, Abmart, China), CHK2 antibody (1∶1,000, CST), cyclinB1 antibody (1∶1,000, CST) or GAPDH (1∶5,000, Kang Chen, China) at 4°C overnight. Secondary antibodies were labeled with HRP (Kang Chen, China) and the signals were detected using ECL kit (Pierce Biotech., Rockford, IL, USA). Subsequently, the images were analyzed by ImageJ 1.43 software. The protein expression was normalized to an endogenous reference (GAPDH) and relative to the control. A relative expression ratio of <1.0 was considered as low expression, while a ratio of >1.0 was considered as high expression [14].

### Cell proliferation assay

Harvested MKN-45 cells (approximately 5 x 10^4^ cells) were seeded into 96-well culture plates. Cellular proliferation was measured at 24 h, 48 h and 72 h post-transfection, respectively, using the Cell Counting Kit-8 (DOJINDO, Japan) according to the manufacturer’s protocol. The absorbance at a wavelength of 450 nm, which shows positive relation to the capacity of cellular proliferation, was determined by a spectrophotometer (E-LIZA MAT-3000).

### Cell cycle assay

Forty-eight hours after transfection, the cells were lifted using 0.25% trypsin and washed in DPBS (Gibco); they were then fixed in 70% ethanol at −20°C for 24 h. For flow cytometric analysis (EPICS XL Beckman Coulter), cells were incubated in RNAse (Fermentas) at 37°C for 30 min, treated with PI (Sigma) and suspended in 300 µl DPBS.

### Statistical analysis

Data were shown as mean ± SD from at least three separate experiments. The significance was analyzed with the Student’s t-test and non-parametric tests (Mann-Whitney U test and Kruskal-Wallis H test). The statistical significance of correlation between the expression of miR-29a and p42.3 protein was calculated by the chi-square test and Spearman′s rank correlation. Statistical analysis was performed using SPSS 13.0 software (IBM, USA) and differences were considered statistically significant at *P* < 0.05.
